# Autonomy support for autonomous motivation in medical education

**DOI:** 10.3402/meo.v20.27951

**Published:** 2015-05-06

**Authors:** Rashmi A. Kusurkar, Gerda Croiset

**Affiliations:** Research in Education, VUmc School of Medical Sciences, Amsterdam, The Netherlands

**Keywords:** autonomous motivation, controlled motivation, self-determination theory, autonomy

## Abstract

**Background:**

Medical students often study only to fare well in their examinations or pursue a specific specialty, or study only those topics that they perceive to be useful in medical practice. The motivation for study in these cases comes from external or internal pressures or from the desire to obtain rewards. Self-determination theory (SDT) classifies this type of motivation as controlled motivation and the type of motivation that comes from genuine interest or personal value as autonomous motivation. Autonomous motivation, in comparison with controlled motivation, has been associated with better learning, academic success, and less exhaustion. SDT endorses autonomous motivation and suggests that autonomy support is important for autonomous motivation. The meaning of autonomy is misinterpreted by many. This article tries to focus on how to be autonomy-supportive in medical education.

**Discussion:**

Autonomy support refers to the perception of choice in learning. Some of the ways of supporting autonomy in medical education are small group teaching, problem-based learning, and gradual increase in responsibility of patients. Autonomy-supportive teaching behavior is not a trait and can be learned. Autonomy support in medical education is not limited to bringing in changes in the medical curriculum for students; it is about an overall change in the way of thinking and working in medical schools that foster autonomy among those involved in education. Research into autonomy in medical education is limited. Some topics that need to be investigated are the ideas and perceptions of students and teachers about autonomy in learning.

**Conclusion:**

Autonomy support in medical education can enhance autonomous motivation of students for medical study and practice and make them autonomy-supportive in their future medical practice and teaching.

Medical students often study so that they can exhibit their knowledge in examinations, pursue their desired specialization, and handle patients in the right manner. If a student perceives that something which needs to be studied does not lead to one of the above outcomes, it is sidelined. This is because the activity of studying is dependent on reasons which are external to the student such as ‘My teacher wants me to study this’ or ‘I am expected to learn this’. On the other hand, if a student studies only to get grades in his examinations or enroll for the specialty he wants, the reasons for studying are still external. This leads to minimal effort from the student which is sufficient for him to achieve his objective. Can this ever lead to deep learning, long-term knowledge, and an attitude of lifelong learning that is expected in the medical profession? Study behavior of the students is dependent on their motivation. What kind of motivation do we want our medical students and doctors to have?

Motivation theories provide us guidance in answering these questions. Self-determination theory (SDT), which is one of these theories, is becoming increasingly popular within medical education. SDT sets store by autonomous motivation, which means that a student learns out of genuine interest or personal value (1, 2). The opposite of autonomous motivation is controlled motivation, which means that a student learns because of external pressures (such as teachers, parents, examinations), internal pressures (guilt or shame), or for rewards (such as grades). Autonomous motivation includes the cases in which a student may not feel genuine interest in an activity, but finds this activity personally important or valuable. For example, a student may study basic sciences not because she finds them genuinely interesting, but because she understands their relevance to clinical practice. Thus, the student perceives involvement in the activity as her own choice. Autonomous motivation, in medical education, has been associated with better learning effort and strategy, better academic performance, and less exhaustion than controlled motivation (3, 4).

How can we keep students autonomously motivated for medical study? SDT emphasizes on the fulfillment of three basic psychological needs: autonomy, competence, and relatedness (1). This article is focused on fulfillment of the need for autonomy in medical education. Autonomy is many times misinterpreted as independence, that is, working without any help, but SDT makes a clear distinction between the two (1). Human beings have an inherent tendency to grow and develop. Autonomous motivation needs to be nurtured not only by providing an autonomy-supportive environment but also by holding back controlling tendency and behavior (1). Autonomy support has been associated with autonomous motivation for medical study and autonomy-supportive medical practice (5). Autonomy-supportive teaching behavior toward medical students has been associated with the students themselves being autonomy-supportive and humanistic in patient care (2). Medical students have also been shown to choose specialties in which they had autonomy-supportive teachers (5). The concept of autonomy support is slowly gaining awareness in medical education (6, 7).

## What is autonomy?

‘Autonomy refers to volition – the organismic desire to self-organize experience and behavior and to have activity be concordant with one's integrated sense of self’ (1). Autonomy is related to striving to make sense of actions and behaviors into a coherent self by visualizing them as self-determined. The opposite of autonomy is control. Autonomy and autonomous motivation lead to greater creativity, satisfaction, and positive well-being (1). Autonomy support in medical education is visualized as providing students with choices in learning, shifting the responsibility of learning to the students, having the internal states of students guide their behaviors, providing optimal challenges, giving constructive feedback that the students can internalize, using autonomy-supportive language, giving emotional support, providing structured guidance, and communicating the value in uninteresting activities (8).

The perception of autonomy is based on three qualities: perceived internal locus of causality, volition, and perceived choice. Reeve describes internal perceived locus of causality as a student engaging in an activity because he wants to do it and not because his teacher wants him to; volition as the feeling of relaxation and absence of pressure to carry out the activity; and perceived choice as the flexibility to take decisions during and related to the activity (9).

SDT distinguishes between autonomy and independence (6). Autonomy does not mean working on one's own without help. Autonomy means working out of one's own choice with no feelings of pressure. It is important to elaborate on this concept with regard to giving undergraduate students and residents autonomy in working with patients. Autonomy in this situation means the student or the resident perceives handling the patient on her own as her choice. She also has the feeling that she has support from the supervisor if she has an opinion which is different from that of the supervisor (10) and if she experiences difficulty in handling the patient. Autonomy with structure is important for self-regulated learning (11). Autonomy without structure can lead to chaos, especially in novice learners. Applying it to the handling of patients by students means that for students just starting their clerkships, there should be different protocols for each type of case from which a student can choose her favored one. Expecting this student to come up with a treatment completely her own is an example of autonomy without structure. Autonomy with structure leads to optimal learning outcomes.

Autonomous motivation is also associated with optimal challenge. If the challenge is just right, the individual experiences what is called ‘flow’. If the challenge is too high and the individual cannot quit the activity in spite of wanting to, he perceives control (1). So, providing autonomy in learning is a skillful task. It demands recurrent questioning of one's methods and adapting one's techniques to the persons and situations involved. Autonomy support can nevertheless be learned (9), the first step being ‘unlearning’ controlling behavior.

## Autonomy support in medical education

Examples of autonomy support in medical education are small group teaching; problem-based learning; gradual increase in responsibility of patients (vertically integrated curricula); opportunities for elective clerkships, to conduct research, for students to enroll in honors programs for higher challenge; the concept of entrustable professional activities (6, 12); and so on.

The need for autonomy is not restricted to students but applies to teachers as well. For teachers to be autonomy-supportive toward students, it is important that they themselves perceive autonomy in their work. Just as students who have autonomy-supportive teachers become autonomy-supportive toward patients, it is possible that teachers who work in autonomy-supportive institutions are autonomy-supportive toward students. Thus, autonomy support in medical education is not limited to bringing in changes in the medical curriculum for students, but encompasses an overall change in the way of thinking and working in medical schools that foster autonomy among those involved in education.

Research into autonomy in medical education is limited (2). The specific topics that need to be explored are the ideas and perceptions of students and teachers about autonomy in learning, the level of autonomy required, the different levels, if any, of required autonomy among students, and the creation of optimal challenges in education to foster autonomy and autonomous motivation.

## Conclusion

Autonomy support in medical education can enhance autonomous motivation of students for medical study and practice and make them autonomy-supportive in their future medical practice and teaching.
